# Changing serotypes of hand, foot and mouth disease in Shanghai, 2017–2019

**DOI:** 10.1186/s13099-022-00485-1

**Published:** 2022-03-21

**Authors:** Linjie Hu, Hairenguli Maimaiti, Lu Zhou, Jie Gao, Yihan Lu

**Affiliations:** 1 Department of Epidemiology, Ministry of Education Key Laboratory of Public Health Safety, School of Public Health, Fudan University, Fosun Tower, 131 Dong An Road, Shanghai, 200032 China; 2 Department of Infection Control, Shanghai Children’s Hospital, Shanghai Jiaotong University, 355 Luding Road, Shanghai, 200062 China

**Keywords:** Enterovirus, Hand, foot, and mouth disease, Serology, Clinical diagnosis, EV-A71 vaccine

## Abstract

**Background:**

Hand, foot, and mouth disease (HFMD) is a common reportable infectious disease that is highly contagious among children in China. This study aimed to characterize the epidemics of HFMD and the serotypes of enterovirus (EV) after the introduction of EV-A71 vaccines in Shanghai, a city in Eastern China.

**Results:**

A total of 2271 HFMD cases were recruited in this study from May 2017 through October 2020. Among these cases, a male-to-female ratio of 1.6:1 was observed, and the cases were mainly in 1–4 years old (63.1%). Children of all ages had a relatively similar time span between the onset of HFMD and the initial medical visit (P = 0.5192). The cases were reported year-round with peaks in the summer (2018 and 2019) and fall (2017), which was consistent with previous epidemics of the reported HFMD cases in the Shanghai municipality. Among the specimens that tested positive for EV (n = 1855), CV-A6 was predominantly detected (71.1%), followed by CV-A16 (14.2%) and EV-A71 (7.0%). Notably, the number of HFMD cases infected with EV-A71 increased in 2019. Furthermore, 9.2% of the cases had comorbidities, mostly convulsion, bronchopneumonia, and pneumonia; however, they were not correlated with the EV serotypes. In addition, 31.2% (709/2271) of the cases were vaccinated with EV-A71 vaccines. The time span differed significantly between the time of vaccination and the onset of the disease across the groups based on whether the onset was before or after vaccination (P < 0.001).

**Conclusions:**

CV-A6 is the predominant EV serotype in the epidemic of HFMD in Shanghai; in addition, CV-A16 and EV-A71 may be moderately prevalent. The changing trends in the presence of EV serotypes contributes to the periodicity of the HFMD epidemic. In addition, the minority of HFMD cases may have comorbidities, regardless of the EV serotype. The use of the EV-A71 vaccine has affected the HFMD epidemic. And serotype-specific protection by the EV-A71 vaccine may promote vaccination in children infected with EV-A71 compared to those infected with non-EV-A71 serotypes, which would further change the epidemic scenario of HFMD.

## Introduction

Hand, foot and mouth disease (HFMD) was first described in 1948. It is an infectious disease, which affects mostly children, and it occurs in preschool children under 5 years old [1]. In recent years, according to the report of the Chinese Center for Disease Control and Prevention, the average annual incidence rate in China has been 139/100,000; the mortality rate has been decreasing year by year. It is at the top of category C infectious diseases in China [2]. In the past 10 years, the incidence rate in the Asia-Pacific region has been increasing [3–8], and China is the main endemic area of HFMD. It has become an urgent issue of both regional and global public health.

The clinical symptoms of HFMD mainly include mild disease due to the herpes virus, and symptoms can be present such as ulcers on the skin or mucous membrane of the hands, feet, oral cavity and other parts of the body, as well as other systemic symptoms such as fever, fatigue, and anorexia. Most of the cases are self-limiting and can be cured within a week with a good prognosis [9]. In addition, some cases of infections with enteroviruses (EVs) that can cause HFMD only manifest with a rash or herpetic angina. A very small number of children can develop severe illness within a short amount of time, with circulatory, respiratory and central nervous system complications [10].

EVs are classified as type A, B, C, D, etc. Among them, 116 serotypes mainly infect humans, and at least 23 serotypes are currently known to cause HFMD [11]. In mainland China, between 2009 and 2016, 70% of severe HFMD cases and 90% of HFMD-related deaths were caused by EV-A71 [12–14]. The HFMD vaccines currently on the market are all EV-A71 vaccines that were approved in December 2015 and December 2016 and are produced by several Chinese companies. The genotypes of the vaccine strains are all C4a genotypes [15, 16].

Therefore, this study is based on the suspected and confirmed cases of HFMD in children admitted to Shanghai Children’s Hospital, describes the epidemiological distribution characteristics, analyzes the changes in the composition of EV serotypes, and compares the main clinical symptoms and comorbidities. This study also evaluates the data regarding the EV-A71 vaccination, to analyze the changing characteristics of HFMD in the post-EV-A71 vaccination era.

## Results

### Epidemiological characteristics of HFMD

**Fig. 1:**
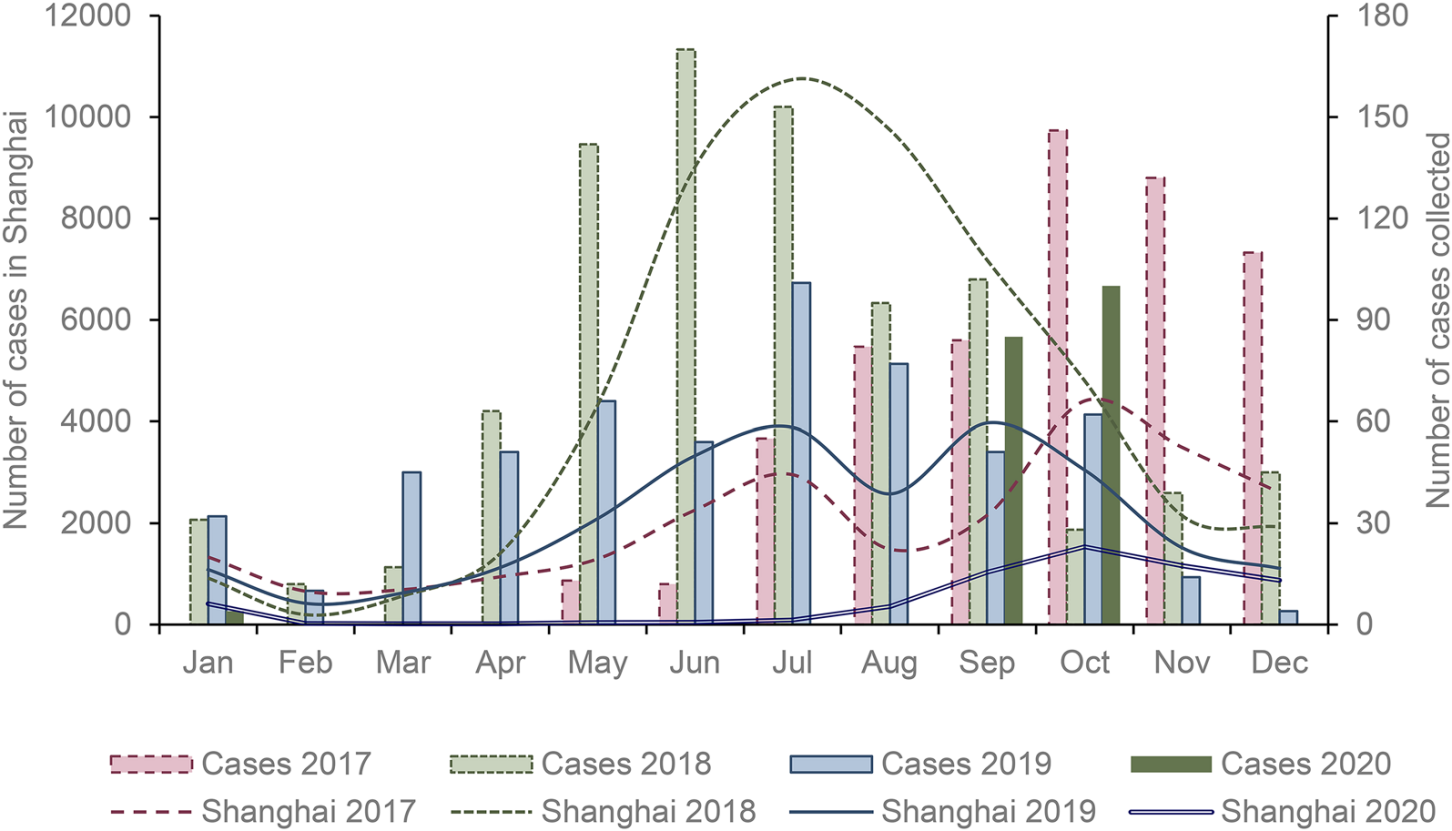
Number of hand, foot, and mouth disease cases collected in present study and in Shanghai from 2017 through 2020

From May 2017 to October 2020, a total of 2271 HFMD cases were collected in this study. As shown in Fig. 1, HFMD occurred all year round. The number of cases began to increase in March and reached a peak in June. The distribution of cases did not show an obvious bimodal distribution. At the same time, we compared the number of HFMD cases reported in the same period in Shanghai. The peak incidence in 2017 was October-November. Since cases were collected in May, this may have had a certain influence on the data in the first incidence peak in 2017. The distribution of the number of cases in 2018 was similar to that in Shanghai, with the incidence peak in June-August and after September the cases started to have a downward trend. The seasonal distribution of the number of cases in 2019 was also similar to the distribution in Shanghai, with two incidence peaks in June-August and September-October. In 2020, the number of cases was smaller from not collecting case numbers due to the coronavirus disease 2019 (COVID-19) pandemic, and cases started to be collected again in September.

Among the cases of HFMD collected in this study, there were 1,400 males (61.6%) and 871 females (38.4%), with a male-to-female ratio of 1.6:1. Of all the patients included, the youngest was 1 day old, and the oldest was 15 years old. The age distribution of patients was mainly concentrated between 1 and 4 years old (63.1%, 1433/2271). Children aged 1–2 years (29.5%) and children aged 3–4 years (17.0%) had the highest prevalence.

A total of 2271 feces, serum and throat swab specimens were collected, and 1855 cases were positive for EV (81.7%). The main detected serotype in the samples was CV-A6 (71.1%, 1319/1855), followed by CV-A16 (14.2%, 263/1855) and EV-A71 (7.0%, 130/1855). In addition, the samples also included CV-A10 (n = 49), CV-A2 (n = 11), CV-A4 (n = 14), CV-A5, (n = 1) CV-A12 (n = 1) and RV- C (n = 4). A total of 12 cases had coinfection with two EVs, mainly CV-A16 with CV-A6 and EV-A71 with CV-A6.

**Fig. 2:**
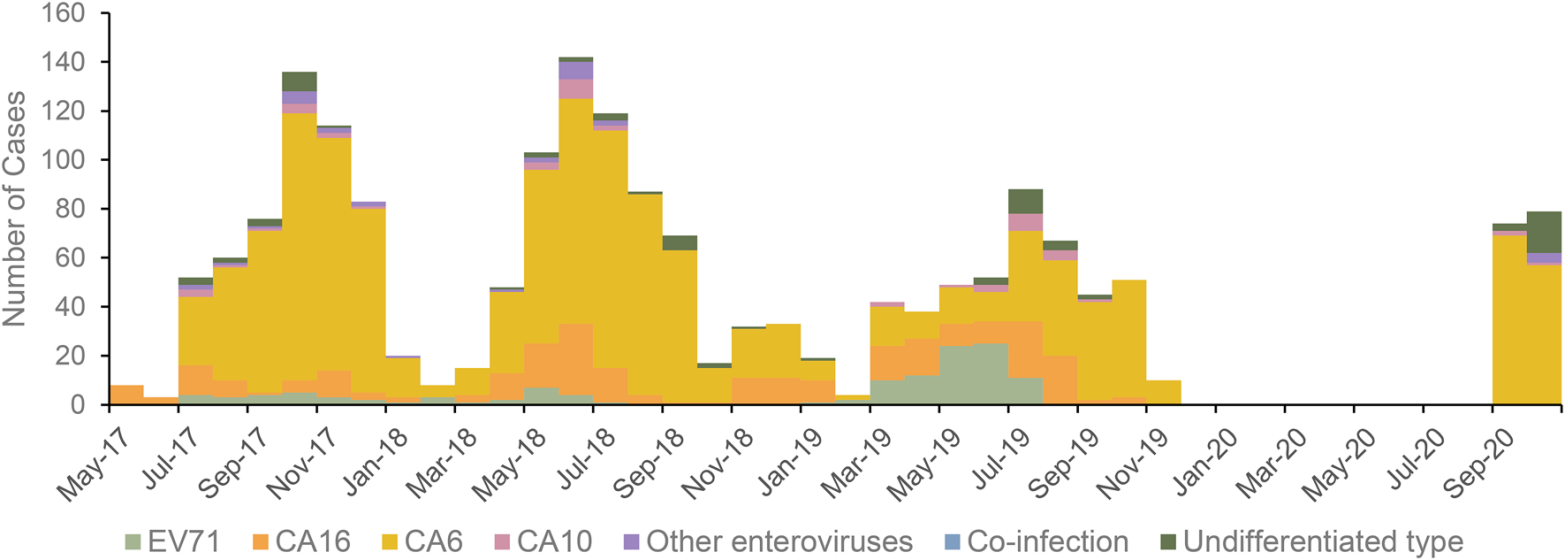
Distribution of serotypes of hand, foot, and mouth disease cases collected from 2017 through 2020

From 2017 to 2020, CV-A6 accounted for the highest proportion of all pathogens detected (Fig. 2). CV-A6 was the predominant serotype found during the high detection months of EV-positive cases from 2017 to 2018, and the number of positive CV-A16 cases increased from February to May 2018. EV-A71 was involved with a substantial increase in cases from January to July 2019 compared with previous years, and the proportion of CV-A16 increased from July to August.

### Clinical and medical characteristics of HFMD

Among all the cases collected in the present study, 1929 cases (85.0%) were diagnosed as HFMD, while 230 cases (10.1%) were diagnosed as herpetic angina. One case was diagnosed as both HFMD and herpetic angina. Some cases were comorbid with other diagnoses (9.2%) (Table 1), mostly including febrile convulsions, convulsions, bronchopneumonia, pneumonia, and bronchitis. These complications were mainly concentrated in the respiratory system (2.6%), digestive system (0.4%) and central nervous system (4.5%). There were no cases that resulted in death. A total of 24 cases were judged to be severe, with comorbidities mainly including digestive system (37.5%, 9/24) and central nervous system (20.8%, 5/24) disorders. Comorbidities that occurred during the same time as the clinical diagnosis were evaluated. Different types of comorbidities (including infectious diseases, digestive comorbidities, nervous system comorbidities, respiratory comorbidities and others) were nonsignificantly correlated with the pathogen serotypes (P = 0.689), but all were significantly related to age (P < 0.001).

**Table 1:** Clinical diagnoses and disease types of cases collected in present study

Clinical diagnosis	Number of cases	Percentage (%)
Hand, foot, and mouth disease	1729	76.1
Herpetic angina	198	8.7
Rash	78	3.4
Hand, foot, and mouth disease and herpetic angina	1	0.0
Hand, foot, and mouth disease and febrile convulsion	47	2.1
Hand, foot, and mouth disease and convulsion	30	1.3
Hand, foot, and mouth disease and bronchopneumonia	26	1.1
Hand, foot, and mouth disease and pneumonia	21	0.9
Hand, foot, and mouth disease and bronchitis	19	0.8
Hand, foot, and mouth disease and digestive system diseases^a^	8	0.4
Hand, foot, and mouth disease and infective fever	4	0.2
Hand, foot, and mouth disease and acute upper respiratory infections	3	0.1
Hand, foot, and mouth disease and other nervous system diseases^b^	6	0.3
Herpetic angina and rash	4	0.2
Herpetic angina and febrile convulsion	7	0.3
Herpetic angina and convulsion	7	0.3
Herpetic angina & bronchopneumonia	1	0.0
Herpetic angina & infective fever	1	0.0
Herpetic angina & digestive system diseases^a^	1	0.0
Rash and acute upper respiratory infections	2	0.1
Rash and bronchitis	2	0.1
Other infectious diseases^c^	27	1.2
Hand, foot, and mouth disease/herpetic angina and other diseases^d^	45	2.0
Total	2267	99.8^e^

^a^Digestive system diseases: diarrhea, abdominal pain, gastrointestinal dysfunction, acute gastritis and acute gastroenteritis

^b^Other nervous system diseases: epilepsy, viral encephalitis, meningitis and facial paralysis

^c^Other infectious diseases: acute upper respiratory tract infection, infectious fever, infectious diarrhea, oral ulcer, oral herpes, mumps, urinary tract infection, bronchitis and chickenpox

^d^Other diseases: acute respiratory infection, thrombocytopenia, traumatic brain injury syndrome, viral skin disease, bronchopneumonia, congenital heart disease, infectious fever, acute upper respiratory tract infection, influenza, dermatitis, angioedema, vehicular accident, febrile convulsion, abnormal pulmonary function, abnormal myocardial enzymes, non-Hodgkin lymphoma, intrahepatic cholangitis, Acute suppurative tonsillitis, urinary tract infection, conjunctivitis, cervical lymphadenitis, benign myositis of the lower extremities, ulcerative stomatitis, thrush, influenza B flow, sepsis, anemia, eczema, premature ventricular contractions, idiopathic thrombocytopenic purpura, chest pain waiting for examination, language development disorders, moderate anemia, thrombocytopenia, bronchitis, congenital heart disease, Congenital upper limb malformation, congenital lower limb malformation, hemangioma, cryptorchidism, congenital heart disease and bacteremia

^e^Among the collected cases, 4 cases were diagnosed as medical consultation and physical examination

**Fig. 3:**
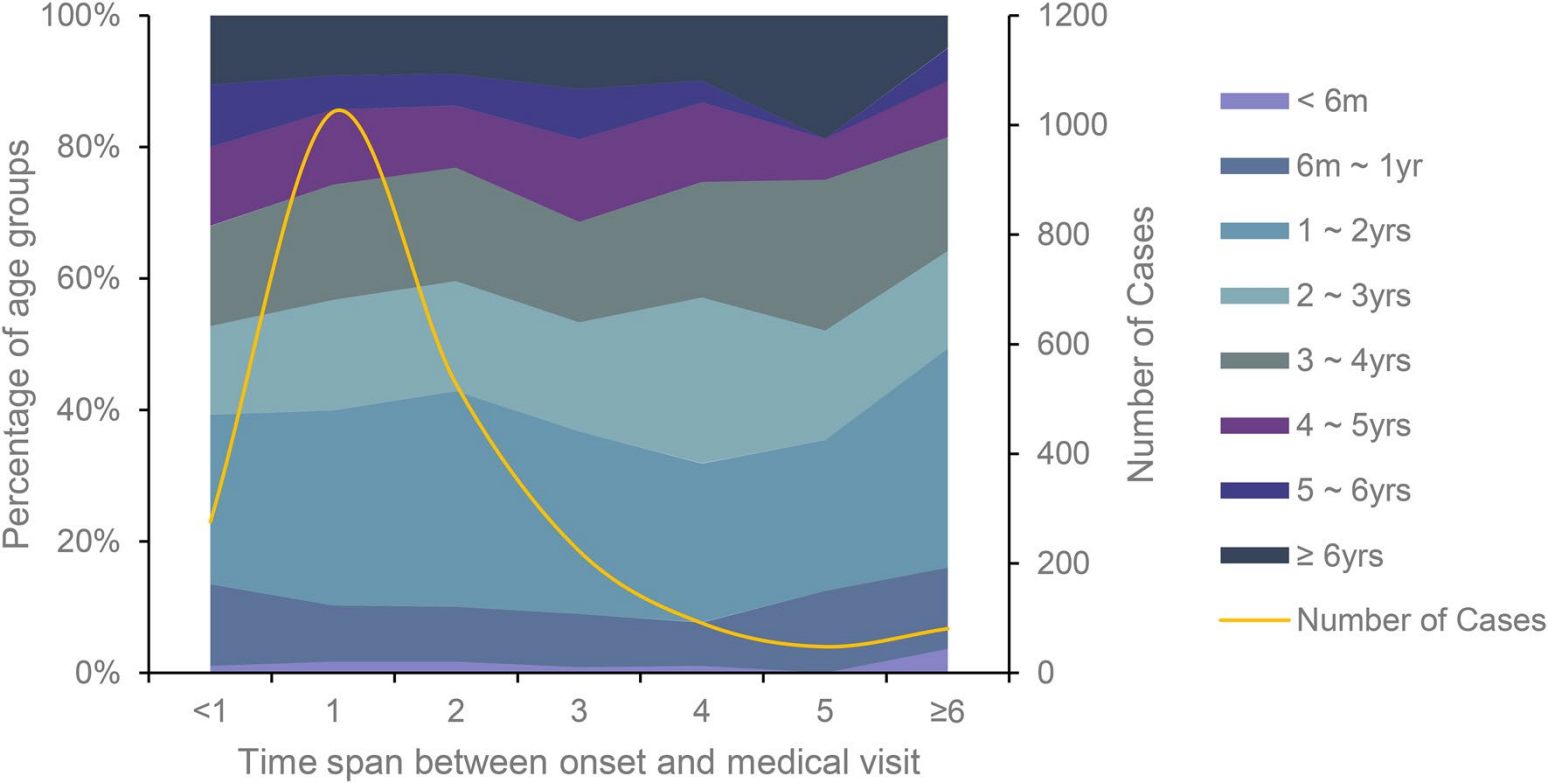
The time span between onset of hand, foot, and mouth disease and medical visit

From 2017 to 2020, the average time from onset to the initial visit for HFMD collected in this study was 1.79 days, while the median was 1 day, and the minimum was zero, which means that the medical visit occurred on the same day as the onset. Meanwhile, the maximum was 33 days. The number of patients who went to a doctor 1 day after the onset of illness was the largest (45.1%) group, followed by patients who went 2 days after the onset (23.2%). The number of patients who went to a doctor within 3 days of the onset accounted for 90.3% of the total number of cases. Children of all ages had a relatively similar time span between the onset of HFMD and medical visits (P = 0.5192), although younger children did not have a significantly longer time span > 6 days (Fig. 3).

### EV-A71 vaccination status

In this study, the EV-A71 vaccination information of 760 cases was obtained through matching. A total of 709 cases had detailed information of the complete vaccination, while the remaining 51 cases had only received the first dose. Comparing the vaccination and the onset time, the onset-vaccination time spans were separated into three types: onset before vaccination, onset during the vaccination process and onset after vaccination. Among the 528 cases onset after full vaccination, there were 1.5% (8/528) severe cases. The eight cases did not comorbid with central nervous system disorders. Among the 76 cases onset after incomplete vaccination (including 25 cases not completing the progress of immunization and 51 cases completing), there were all mild cases. A total of 156 cases had onset before vaccination, with 1.3% (2/156) severe cases, including one severe case with central nervous system disorders.

In the present study, we eventually found 5.7% (130/2271) cases to be EV-A71 positive, with one severe case. Among cases onset after at least one-dose vaccination, the percentage of EV-A71 positive cases was 6.1% (37/604), while the percentage among cases onset before vaccination was 6.4% (10/156).

**Fig. 4:**
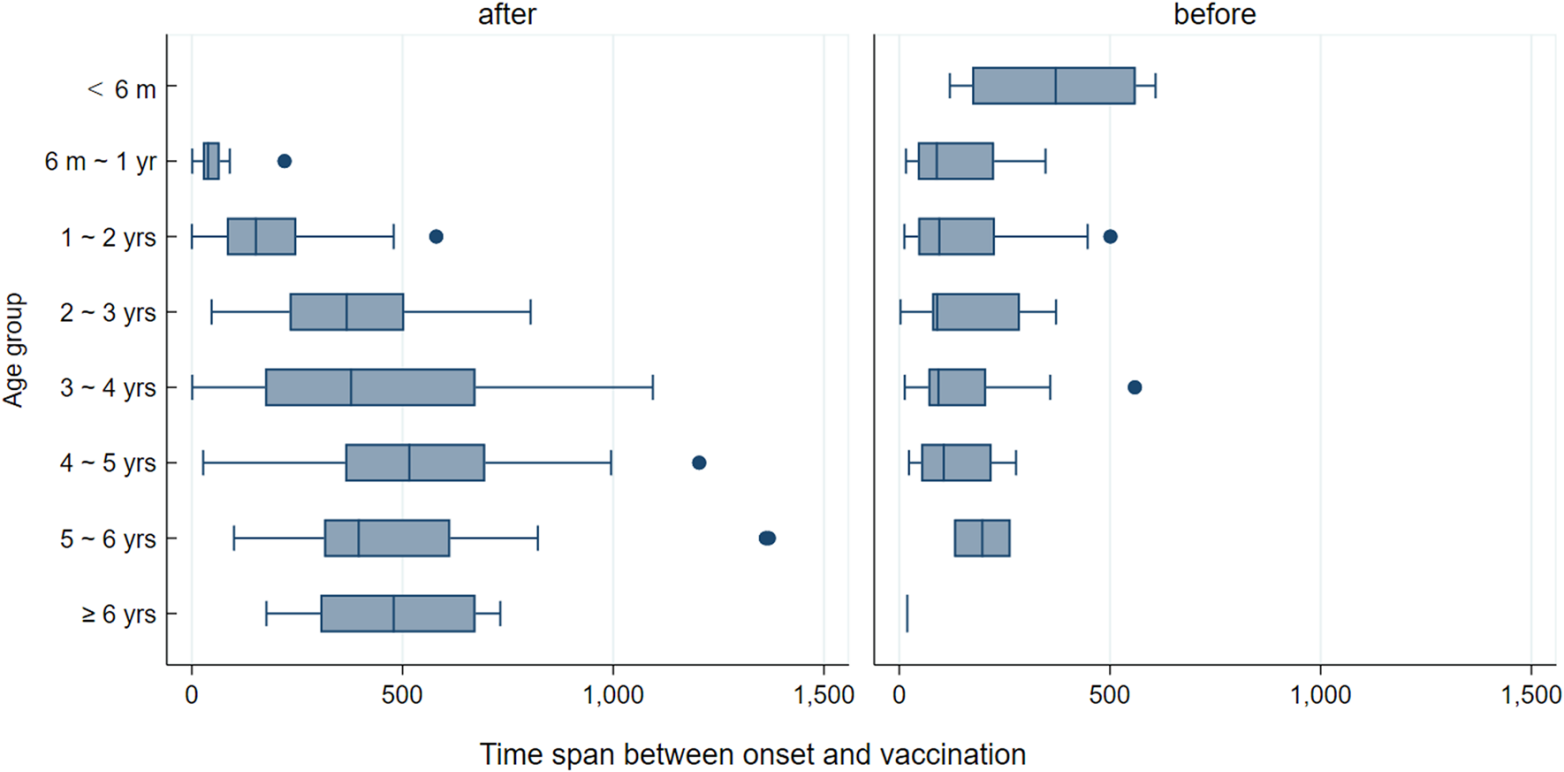
Onset-vaccination time spans grouped by age groups

**Fig. 5:**
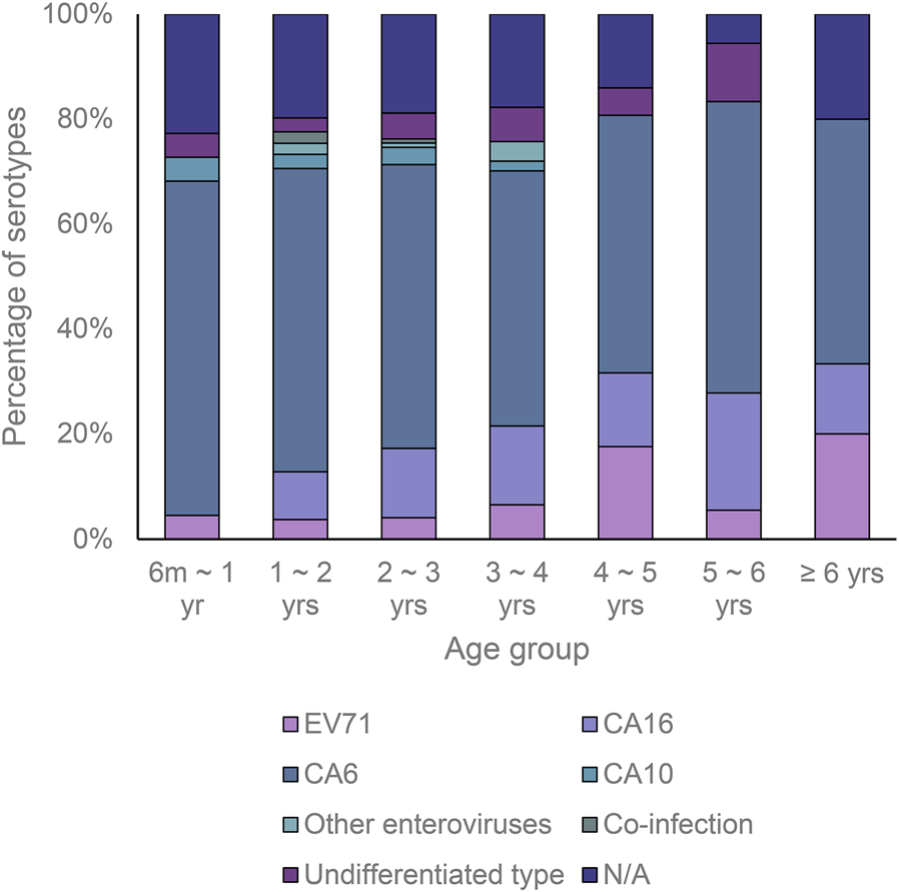
Distribution of serotypes of hand, foot, and mouth disease cases onset after vaccination

Due to the small number of cases with disease onset occurring between the first and second vaccination, only patients with an onset before and after vaccination cases were considered. The specific distribution is shown in Fig. 4; the serotype distribution of case onset after vaccination is shown in Fig. 5. There was a significant difference in the time span between the onset and the closest vaccination time between the groups before and after vaccination (P < 0.001). The average onset-vaccination time spans (Table 2) were not significantly different between the two statuses. The average time span of the 35 EV-A71 positive cases onset after full vaccination was 383.0 ± 242.8 days, while 322.6 ± 248.9 days of other serotypes positive cases. There was no significant different (P = 0.177).

**Table 2:** Average time spans of cases onset before and after vaccination

Vaccination-onset status	Serotype	Proportion (%)	Average time span (days)^a^
Onset after vaccination	All tested	–	326.6
Onset after vaccination	EV-A71	6.6 (35/528)	383.0
Onset before vaccination	All tested	–	152.0
Onset before vaccination	EV-A71	6.9 (9/130)	116.8

^a^ The average time span between onset and closet vaccination time

## Discussion

This study found that the seasonal distribution of HFMD in Shanghai may be affected by the different epidemic serotypes from 2017 to 2020. The obvious incidence peak was mainly caused by the incidence fluctuation of CV-A6. Previous studies have shown that HFMD has obvious seasonality, and there are generally two peaks of incidence each year, namely, the main peak in the spring and summer and the secondary peak in the autumn [17]. Studies have also shown that the half-year periodicity of HFMD was in South China, while the single peak in June appeared in North China annually [1]. However, in this study, bimodal and unimodal periodicities occurred in different years. We found that the peak was higher in the autumn of 2017 (September-November), while the summer peak (June-August) was mainly seen in 2018. Due to the impact of the COVID-19 pandemic, cases were only collected in September and October 2020. The distribution and trends may be different, so we did not discuss them here. After further exploring the spectrum of serotypes, it was found that the main difference between 2017 and 2018 was the peak of CV-A16 in the summer. In 2019, the peak of EV-A71 appeared around May, and the peak of CV-A16 appeared in March-April and July August. The change in the serotype spectrum might be due to interference caused by changing predominant pathogenic EV serotypes and the launch of the EV-A71 vaccine, which complicates the HFMD periodicity. It was reported that the annual peak of CA-V16 appeared earlier than that of other enteroviruses [1]. The change in the serotype and the immunity gained from an infection with HFMD might be related to the periodic epidemic [18]. Since 2013, the proportion of EV-A71 serotypes among the pathogens of HFMD has declined in Shanghai and other provinces of China [19–23]. Based on this data, further studies are needed to distinguish the contribution of the EV-A71 vaccine and natural epidemics to the changing epidemic characteristics of HFMD.

The proportion of male children in this study was slightly higher than that of females. Studies have confirmed that the infection rates in males and females were similar, but males were more likely to have symptoms, more likely to have diffuse infections and more likely to need medical assistance [24, 25]. This study also included a small number of cases that had herpetic angina. The early clinical manifestations of some children with HFMD were similar to the symptoms of herpetic angina, and the two overlapped in etiology, which was easily confused in clinical diagnosis [26]. Furthermore, some cases were comorbid with other symptoms (9.2%), mainly in the respiratory, digestive and central nervous systems. Although our study did not find a correlation between the serotypes and comorbidities, since severe HFMD often leads to more serious disease outcomes, further research may provide evidence for preventive strategies. In addition, in this study, patients with HFMD had an average of 1.79 days from onset to treatment. The findings of the Chinese Center for Disease Control and Prevention showed that the time span between onset and diagnosis of severe cases had a significant impact on whether the patient died [27], prompting patients and their parents to have a medical visit as soon as possible after the onset, especially those with severe manifestations.

Since Shanghai began to vaccinate the EV-A71 vaccine in 2017, studies have shown that the efficacy of the vaccine against EV-A71-related HFMD was similar in children aged 6–23 months and in children aged 24–71 months [28]. The proportions of serotypes in each age group in the study were also basically similar. Our study found that the age of patients at vaccination was concentrated between the ages of 6 months to 2 years, and the onset-vaccination time span of the lower age groups was longer among the cases which had their disease onset before vaccination, especially in patients less than 6 months of age. This was presumed to be related to the age restriction for vaccination; the time span was longer in the upper age group among the cases which had disease onset after vaccination, which may be related to the average vaccination age in present study. The present study and the previous studies found that EV-A71 is no longer the most predominant HFMD serotype in Chinese mainland, and we can affirm the efficacy of the vaccination in controlling EV-A71 epidemic, although there are still EV-A71 positive cases. However, we could not find the evidence of cross immunity across genotypes of EV.

Cases that developed the onset after vaccination were classified as EV-A71, CV-A16, CV-A6, CV-A10, CV-A2, CV-A4, RV-C serotypes and other serotypes. In this study, patients infected with EV-A71 received post onset vaccination earlier than patients with other serotypes, probably due to the specific protectivity of the vaccine for this serotype. The proportion of cases still infected with the EV-A71 serotype after vaccination was lower, but the average time span between vaccination and onset was shorter than the vaccine protection period, which was proven to be more than 2 years [29]. It may be that the individual has milder symptoms after vaccination and is not inclined to seek medical treatment. The study found that patients who completed the full vaccination were less likely to have central nervous system comorbidities, while the small sample size limited this result to allow for a better statistical test. It is worth noting that although the number of cases of EV-A71 infection was small after two doses of the EV-A71 vaccine, it is necessary to arouse public health concern about infection and comorbidity after vaccination considering the limited number of cases included in this study. The results of this study showed that there was a small increase in cases of the EV-A71 serotype in 2019. This may indicate that although EV-A71 is not currently the main serotype of HFMD, we should consider its recurrent trends. For more severe clinical symptoms, children need to be vaccinated with the EV-A71 vaccine to avoid an epidemic. This study and previous studies have found that there were multiple infections of HFMD, and there were even cases of infection with the EV-A71 type and then subsequent infections with other types such as CV-A16 [30], which indicates that more scientific research efforts are urgently needed in the development of multivalent vaccines.

Some limitations are present in the current study. First, the sample size collected by the study was small. However, the trend of cases collected in this study in the past 4 years was broadly consistent with the trend of reported cases in Shanghai. The detection of all serotypes of EV in this study was more accurate than the reported data that only required the detection of EV-A71, CV-A16 and other serotypes, which can reflect the seasonal trend of the predominant serotypes. Second, approximately 10% of the cases collected in this study had herpetic angina, and this is currently not a legally reported infectious disease in China. However, its pathogenic spectrum (also CV-A6) might have a certain impact on the overall observation results. However, according to the diagnostic guidelines of HFMD in China, some of these cases with an overlapping etiology can also be diagnosed as HFMD. In fact, the diagnostic difference between the two may not be clear. Third, we have not sequenced the EV-A71 positive specimens in this study. It results in disability to confirm the genotypes of EV-A71 and raises the question of assessing the efficacy of vaccines on different genotypes. In addition, in the present study, we considered all acquired diagnoses as comorbidities of HFMD. It may not accurately reflect the actual situation of HFMD comorbidity in this study. There may be recall bias in the study due to caregivers’ memory regarding the date of onset.

## Conclusions

This study found that CV-A6 was the predominant serotype in the epidemic of HFMD in Shanghai, while CV-A16 and EV-A71 might be moderately prevalent, which shaped the periodicity between 2017 and 2019. In addition, less than 10% of HFMD cases had comorbidities, regardless of the serotype. The immunization program for EV-A71 vaccines has affected the prevalence of HFMD. However, the characteristics of EV-A71 vaccination differed across the age groups of children. Serotype-specific protection by the EV-A71 vaccine might promote vaccination for children infected with EV-A71 compared to those infected with non-EV-A71 serotypes, which would change the scenario of HFMD. Further strengthening EV-A71 vaccination and the development of multivalent vaccines are warranted.

## Methods

### Study participants

A total of 2271 patients with HFMD in Children’s Hospital of Shanghai were included from 2017 to 2020. The diagnosis of HFMD was made after comprehensively considering the epidemiological history, clinical manifestations and etiological examinations. The diagnosis of all cases complied with the Chinese guidelines for the diagnosis and treatment of hand, foot and mouth disease [2]. Meanwhile, the reported cases of HFMD in Shanghai were collected, and the epidemic data were from the Shanghai Municipal Health Commission.

Suspected cases of HFMD were defined as patients who had papules or vesicular rashes on the hands, feet, oral cavity or buttocks, with or without fever. Confirmed cases were defined as cases of EV infection (including EV-A71, CV-A16, CV-A6 and other serotypes) confirmed by laboratory evidence, such as RT-PCR, real-time RT-PCR, or virus isolation. Because of the clinically overlapping symptoms and the similar etiology with HFMD, this study included all suspected and confirmed cases of HFMD, including cases diagnosed as herpes angina and other highly suspected or laboratory-detected cases of EV infection. For the convenience of explanation, we uniformly defined the collected cases as HFMD cases.

### Data and specimen collection

The study collected basic information on the included patients, including sex, date of birth, date of onset, date of diagnosis, and the clinical diagnosis. At the same time, we used the patient number to match the vaccination data from the Shanghai Municipal Immunization Planning Information System to obtain the date of EV-A71 vaccination. Throat swabs, feces and serum specimens of children with HFMD were collected, transported at 0–4 °C and stored at − 80 °C.

### Laboratory detection

The Qiagen Viral RNA Extraction Kit (QIAGEN, Germany) was used to extract viral RNA. The extraction process was carried out according to the manufacturer’s instructions. A CFX 96 real-time fluorescence quantitative polymerase chain reaction system (BioRad, Hercules, CA, USA) was used to amplify the viral RNA and detect and analyze the fluorescence signals. In this study, EV universal primers were examined by using a Fluorescence Quantitative Polymerase Chain Reaction (qPCR) Kit (BioGerm, Shanghai, China). Those patients who were positive continued to be tested and classified as EV-A71, CV-A6, CV-A10, or CV-A16. The negative specimens continued to be detected by a Reverse Transcription-Polymerase Chain Reaction (RT-PCR) Kit (TAKARA, Japan) and were then sequenced to determine their serotypes. When the Ct value was > 38 or if pathogens were not detected, the specimens were confirmed as negative. The specimens with an S-shaped amplification curve and a Ct value ≤ 35 were confirmed to be positive. If the amplification curve was S-shaped and if the Ct value was between 35 and 38, the experiment was repeated; then, if the amplification curve was still S-shaped and if the Ct value was between 35 and 38, it was judged as positive. Otherwise, it was negative.

### Statistical analysis

We included all of the cases in the analysis and grouped the patients by age: less than 6 months, 6 months to 1 year, 1–2, 2–3, 3–4, 4–5, 5–6 years and 6 years and older. Patients with HFMD, whether suspected or confirmed, were classified as severe if there were any neurological complications (aseptic meningitis, encephalitis, encephalomyelitis, acute flaccid paralysis or autonomic nervous system disorders) and/or cardiopulmonary complications (pulmonary edema, pulmonary hemorrhage, or cardiopulmonary failure); otherwise, they were classified as mild cases, as documented elsewhere [2]. We used descriptive methods to analyze the epidemiological and clinical characteristics of the HFMD. Spearman’s correlation model was applied to test the correlation between the comorbidities and the pathogen serotypes, as well as the age groups. It was also used to test the correlation between the age group and the time span from onset to the initial visit. Wilks’ lambda of discriminant analysis was used to test the difference in the time spans among the groups based on the vaccination status. Student’s t was used to test the difference in the time span between EV-A71 cases and cases of other serotypes onset after vaccination. IBM SPSS 20.0 (IBM, Somers, NY, USA) and Stata 16.0 (Computing Resources Center, Santa Monica, CA, USA) were used for the statistical analysis, and a P < 0.05 indicated that the difference was statistically significant. Excel 2016 (Microsoft, Redmond, WA, USA) and Stata 16.0 were used to draw statistical graphics.

## Data Availability

The datasets generated during the current study are available from the corresponding author Dr. Yihan Lu on reasonable request.

## Abbreviations

HFMD: Hand, foot, and mouth disease
EV: Enterovirus
COVID-19: Coronavirus disease 2019
qPCR: fluorescence quantitative polymerase chain reaction
RT-PCR: Reverse transcription-polymerase chain reaction
